# Preliminary findings from a formative evaluation of the Indigenous peer support specialist train-the-trainer manual: a culturally grounded approach to recovery in American Indian and Alaska Native communities

**DOI:** 10.3389/fpubh.2026.1833928

**Published:** 2026-09-03

**Authors:** Tassy Parker, Allyson Kelley, Norman Cooeyate, Marcello Maviglia, Donald Hume

**Affiliations:** 1 University of New Mexico Health Sciences, Center for Native American Health, Albuquerque, NM, United States; 2 Allyson Kelley and Associates PLLC, Sisters, OR, United States

**Keywords:** Alaska Native, American Indian, curriculum, Indigenous recovery, peer recovery support specialist, pilot study

## Abstract

**Introduction:**

American Indians and Alaska Natives (AIANs) are rich in culture, heritage, language, and resilience. Chronic underfunding, limited healthcare infrastructure, and barriers to quality health services contribute to some of the most severe health disparities in mental health and substance use disorders among AIAN populations.

**Methods:**

This formative evaluation aims to document the process and early implementation feedback on an Indigenous Peer Support Specialist Train-the-Trainer Manual (IPSSTTM) designed for use across Indigenous, community, clinical, and university settings. The curriculum was piloted from 2024 to 2026 in collaboration with the University of New Mexico Center for Native American Health (UNM CNAH), a peer recovery support worker, and an external community-based recovery organization (AKA). Initial drafts were developed, reviewed for content, culture, and applicability, and compiled into a 143-page manual. A culturally centered, Indigenous, mixed-method, Two-Eyed seeing approach was employed to gather and analyze pilot feedback on the curriculum. Various data types were used to explore meaning and identify areas for improvement within Indigenous contexts.

**Results:**

This formative evaluation suggests the curriculum is an innovative, culturally grounded intervention that combines Indigenous healing practices with recovery needs. By centering Indigenous worldviews, building local capacity and workforce, and promoting sustainable knowledge transfer, this initiative may support behavioral and mental health equity. It strengthens Indigenous communities to address substance use and mental health challenges with resilience, cultural reverence, and sustainability.

**Discussion/Conclusion:**

Early feedback from participants underscores the importance and urgency of providing culturally constructed recovery support services in Indian Country and that the IPSSTTM is a promising approach.

## 1 Introduction

American Indian and Alaska Native (AIAN) communities represent 9.7 million people in the United States and include 575 federally recognized Tribal Nations (1). As America’s First Peoples, AIANs possess a rich history, culture, language base, and resilience that have helped them endure war, genocide, forced removal from homelands, abuse, violence, and persistent oppression and marginalization by mainstream colonial America. Although resilient and culturally rich, AIANs have the lowest life expectancy in the United States (2). Chronic underfunding, limited infrastructure of health systems, and barriers to quality mental and behavioral health care create conditions where AIAN people experience some of the most severe health disparities from mental health and substance use disorders. AIANs experience the highest mortality from alcohol, opioids, and polysubstance use, and die from alcohol-related factors at a rate that is five times higher than that of whites in the United States (3). AIANs are less likely to receive treatment medications for opioid use disorder and less likely to be diagnosed with comorbid psychiatric conditions, suggesting gaps in referral sources, service utilization, and clinical management of AIAN SUD (4). Compounding these conditions is the overreliance on Western behavioral health models and concepts of wellness that prioritize treating disease rather than the whole person or community.

From an Indigenous perspective, recovery is rooted in a communal approach, with support systems available throughout an individual’s life cycle to support their healing and recovery. However, the acceptance of this approach can pose challenges for Western-trained practitioners, as much of the Indigenous empirical evidence is underrepresented in peer-reviewed academic literature and evidence-based initiatives. Offering an Indigenous-specific approach to recovery is necessary because it is grounded in the life experiences, values, and beliefs of communal recovery within Indigenous cultures. While most Peer Recovery Support (PRS) literature differentiates between mental health and substance use issues, most Indigenous approaches do not. In this work, we avoid diagnostic labeling and refer to the notion of distress, which includes psychological, social, cultural, and spiritual domains. This aligns with Indigenous practices that view distress holistically and in context. We propose that the Indigenous Peer Support Specialist Train-the-Trainer Manual (hereafter referred to as the Manual) offers a culturally constructed approach to the limitations of Western-based recovery options for persistent distress that contributes to the lower life expectancy of AIAN people. See Table 1 for relational concepts of recovery from Tribal language and teachings.

**Table 1:** Relational concepts of recovery from tribal language and teachings.

Tribe Name^	Term	Teachings
Seneca	Orenda	Deep reflective thought leads to knowledge and spiritual growth, revealing our purpose on earth.
Zuni Pueblo	Hon i:wichemanan:wa	We will love one another
Navajo	*Hózhóójí*	The Beauty Way
Shoshone	Natsu	Healing
Crow	Bishtataáwahtchishik	Healing/I am healing
Cheyenne	Nápévomóhtahe	I’m feeling good. Implies a state of physical and mental well-being.

^^^Note that the authors contributed to or consulted with Tribal members who shared words and definitions based on their own understanding and teachings.

### 1.1 Theoretical framework and architecture of the IPSSTTM

The theoretical architecture of the IPSSTTM is rooted in a coherent, fully Indigenous paradigm of healing that stands apart from Western behavioral health frameworks. Rather than adapting or hybridizing Western constructs, the IPSSTTM articulates a complete epistemological system grounded in Indigenous relationality, cyclical understandings of wellness, community-defined evidence, and cultural continuity as the primary mechanism of recovery.

#### 1.1.1 Relationality as the foundation of healing

Indigenous worldviews understand that relationality is the foundation of healing. Wellness is understood as emerging from the quality of relationships and the knowledge that stems from them, healing the individual, family, community, land, ancestors, and future generations. Distress is not conceptualized as an individual pathology but as a disruption in relational balance (5) and the power (knowledge) applied to developing and maintaining the balance. Indigenous peer support specialists, therefore, serve as relational anchors who embody cultural teachings, model communal responsibility, and facilitate collective meaning-making. Their authority derives from lived experience, cultural grounding, and relational accountability rather than clinical training.

The newly developed Indigenous Cycles of Healing and Recovery Model conceptualizes recovery as a dynamic, recursive process shaped by context, relationships, and cultural teachings (6). This cyclical model challenges the linear “stages of change” framework that dominates Western behavioral health. Linear models assume predictable progression, individual agency, and measurable behavioral outcomes. In contrast, this model recognizes that healing unfolds in relation to community responsibilities, cultural obligations, and historical context. This model is grounded in community-defined evidence, knowledge generated through lived experience, cultural teachings, and practice-based wisdom accumulated over generations. This epistemological stance challenges Western evidence hierarchies that privilege randomized trials and clinician-driven outcomes (7). In Indigenous contexts, evidence is relational, contextual, and validated through community consensus rather than external evaluation.

Cultural continuity, including language, ceremony, kinship structures, and traditional practices, is understood as the primary mechanism through which healing occurs. Cultural teachings are not added to a pre-existing clinical framework; they constitute the framework itself. This emphasis on cultural continuity also serves as a corrective to the historical erasure of Indigenous healing systems through colonization and the imposition of biomedical authority, or the power of the medical profession (8). By grounding recovery in relationality, cyclical healing, community-defined evidence, and cultural continuity, this model reframes distress as a communal and historical experience rather than an individual pathology. This theoretical stance aligns with global critiques of psychiatric overreach and affirms the need for culturally grounded, community-driven approaches to wellness (9, 10).

## 2 Materials and methods

### 2.1 Indigenous epistemologies of healing and foundations of the model

A central theoretical concept of the Indigenous Peer Support Specialist Train the Trainer Model (IPSSTTM) is the recognition that Western behavioral health systems have increasingly medicalized distress, reframing complex social, cultural, and historical experiences as individual psychiatric disorders. Included in the Manual is a section that highlights how this process (rooted in diagnostic expansion, pharmaceutical marketing, and structural pressures within healthcare systems) has contributed to the chronicity of distress rather than its resolution.

Medicalization operates through several mechanisms, including the reduction of lived experience to symptoms and the obscuring of relational, cultural, and historical contexts (11); pathologizing normative responses to trauma, oppression, and social suffering (12); overreliance on psychotropic medications, often without adequate attention to long-term risks or the absence of evidence for many diagnostic categories (9, 10); and marginalization of community-based, relational, and cultural healing practices- which are often dismissed as “non-evidence-based” despite strong practice-based evidence in Indigenous communities (8).

For Indigenous Peoples, medicalization is inseparable from colonial histories. Western psychiatric frameworks have historically misinterpreted Indigenous relational worldviews, grief practices, and communal responsibilities as pathology, leading to misdiagnosis, coercive treatment, and erosion of cultural authority (8). The biomedical model’s dominance has also contributed to structural inequities, including workforce shortages, fragmented care, and the displacement of Indigenous healing systems.

The IPSS-TTM responds directly to these concerns. By centering Indigenous epistemologies, the manual de-medicalizes emotional distress, reframing it as a relational and communal experience rather than an individual “disorder.” This approach aligns with global critiques of psychiatric overreach, which argue that emotional suffering is often better understood through social, cultural, and moral frameworks than through diagnostic labels (7).

### 2.2 Tribal innovation and the emergence of peer recovery support models

The IPSS-TTM emerged from decades of tribal innovation in response to structural gaps in behavioral health systems. AIAN communities continue to experience disproportionate burdens of substance-related morbidity and mortality, driven not by individual pathology but by chronic underinvestment, workforce shortages, and the historical imposition of Western biomedical models (8, 11) based on physical reductionism, objective diagnoses, and targeted interventions that fail to reflect holistic Indigenous worldviews and the importance of trust and transparency in AIAN communities (13). In this context, Tribes have long developed their own solutions to substance-related morbidity and mortality that are practice-based, culturally grounded, and community-defined.

The Manual was culturally constructed with the following elements:

- Lived experience and community priorities (rather than academic or clinical theory)
- Practice-based evidence, accumulated through decades of peer support, cultural teachings, and community healing
- Iterative feedback from peer specialists, community reviewers, and early implementation groups
- A train-the-trainer structure designed to build local capacity, ensure sustainability, and protect cultural fidelity

This developmental process aligns with broader Indigenous behavioral health initiatives, such as the Indigenous Recovery Planning (IRP) intervention, which demonstrated the feasibility and effectiveness of training non-specialist community members to deliver culturally grounded recovery programs (14).

Data from the Substance Abuse and Mental Health Services Administration (SAMHSA) indicates that peer support services are a successful mechanism for working with individuals who are in recovery (15). Peer support can extend beyond the clinical setting, providing individuals with greater control, empowerment, social support, and hope in their recovery. Research suggests that peer recovery support services result in reduced substance use, increased treatment retention, increased satisfaction with treatment, reduced relapse rates, and reduced use of emergency services and involvement with the criminal justice setting (15).

In response to unmet needs and systems-level limitations, Tribes developed their own solutions to foster healing, resilience, and wellness outside or alongside state and federal programs. The Peer-to-Peer approach (also known as Peer Mentoring, Peer Coaching, and/or Peer Recovery Support Specialist) has emerged as a successful tool in Tribal communities. Peer support is a strengths-based response to identifying gaps in mental and behavioral health support, which highlights reflective recovery practices, mutual aid, and culturally relevant peer support (16–21). Peer support involves giving and receiving help based on key principles of respect, shared responsibility, and mutual understanding of what is helpful. It aims to deepen understanding of another’s situation through shared emotional and psychological experiences (22). Peer support providers can serve many roles in supporting individuals with psychiatric conditions or those who are in addiction recovery. They can lead education and support groups and act as a bridge connecting people to services as they transition from hospitals or jails back into the community. Instead of viewing peer support as just an addition to clinical care, this approach centers Indigenous values, lived experience, and relational healing as fundamental mechanisms of recovery for both individuals and communities. Prior research and publications demonstrate how Tribal communities, organizations, and universities are creating Indigenous models of peer recovery support (21) and suggest that these models are more culturally responsive than Western or non-Indigenous healing approaches (17, 18, 23). Evaluations of these PRS models reveal common themes in Indigenous settings, including a deep understanding of culture, values, norms, and healing practices. When these are integrated into PRS approaches, they are often more desirable and effective for the person in need (18).

### 2.3 Applying Indigenous healing concepts in a peer-led recovery intervention

The conceptual framework for developing the curriculum drew on teachings from Indigenous grandfathers, existing peer recovery support literature, and cultural adaptations of healing practices (24). The Indigenous Peer Support Specialist Train-the-Trainer Manual is grounded in a distinctly Indigenous worldview of healing that centers relationality, communal responsibility, and cyclical processes of wellness. This strengths-based approach and cultural flexibility promote self-determination and self-governance at the local level.

In Indigenous knowledge systems, healing is inseparable from land, kinship, spirituality, and intergenerational continuity. Recovery is not conceptualized as an *individual* clinical outcome but as a relational, *community*-anchored process that unfolds in multiple stages across the life cycle. This orientation contrasts sharply with Western behavioral health frameworks, which typically emphasize individualism, linear models of change, and symptom-based metrics (8).

**Figure 1:**
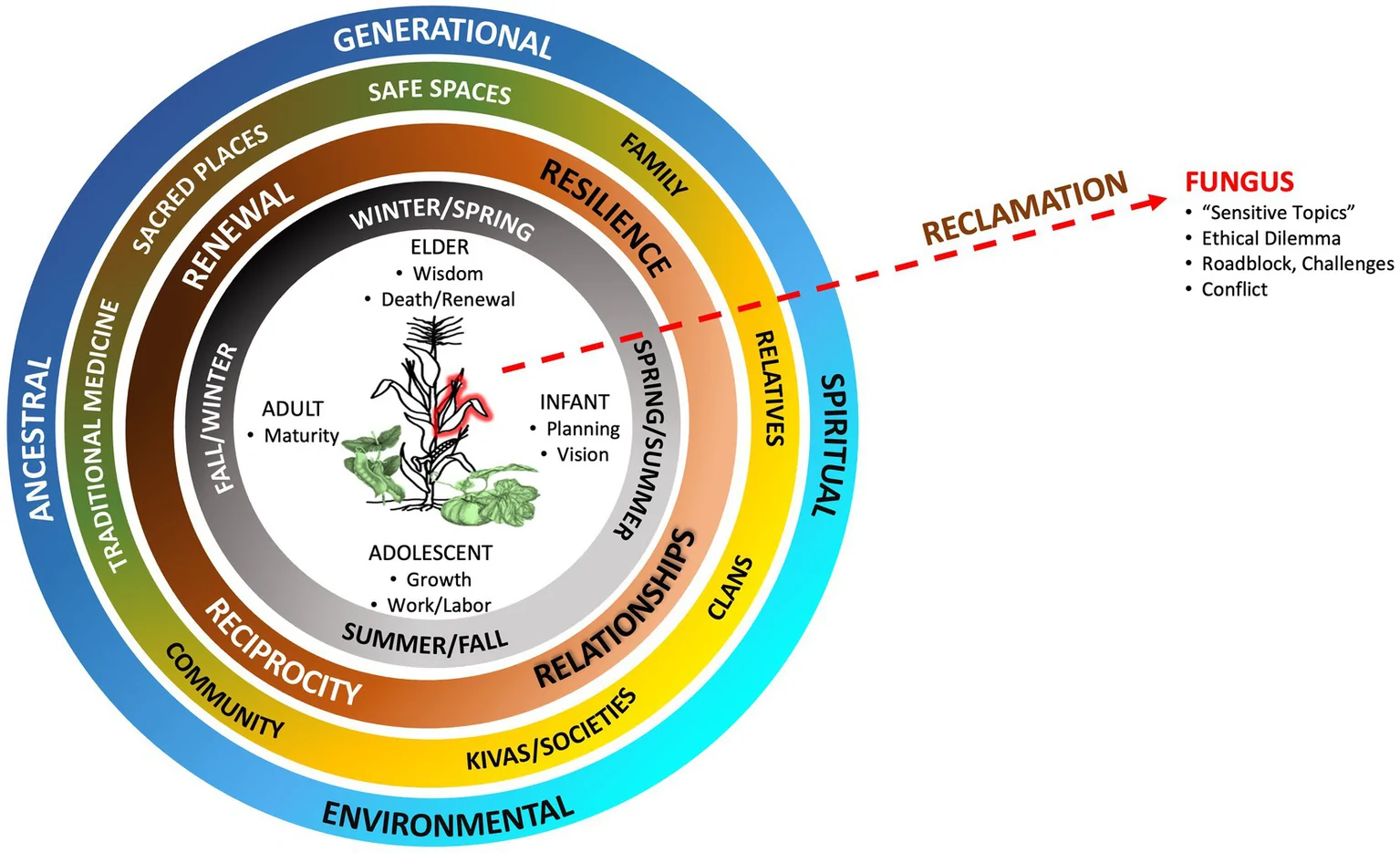
Indigenous cycles of healing and recovery model.

The Indigenous Cycles of Healing and Recovery model (Figure 1) conceptualizes healing as cyclical, fluid, and responsive to community and ecological context. It is an explicit alternative to the linear “stages of change” that dominate Western behavioral health (6). Within this framework, peer specialists are not paraprofessionals delivering standardized techniques; they are cultural knowledge holders, relational anchors, and facilitators of community-based healing. Their authority is derived from lived experience, cultural grounding, and relational accountability rather than clinical training.

Of note, the Manual is not based on a hybrid model blending Indigenous and Western methodologies. Western behavioral interventions like Motivational Interviewing (MI), cognitive-behavioral approaches, or biomedical recovery frameworks are grounded in assumptions that do not align with Indigenous worldviews. MI, for example, is built upon *individual* ambivalence, clinician-guided elicitation of motivation, and a dyadic provider-client structure (25). These assumptions contrast sharply with Indigenous recovery processes, which emphasize *collective* responsibility, shared meaning-making, and healing through community relationships rather than individual behavioral change.

The Manual does not “Indigenize” Western constructs; it replaces them with Indigenous constructs. Talking Circles, peacemaking circles, and community-defined pathways to recovery serve as the primary mechanisms of healing; they are not adaptations of clinical interviewing or motivation-shifting strategies. This preserves the epistemic integrity of Indigenous knowledge systems and avoids the dilution that often occurs when Western frameworks are retrofitted with cultural elements.

### 2.4 Formative evaluation of the manual

This formative evaluation aims to document the process and early implementation feedback of an IPSS-TTM for use in a variety of Indigenous, community, clinical, and university settings. The COVID-19 pandemic exposed and worsened the severe opioid crisis among AIAN people, who saw a disproportionate rise in both fatal and non-fatal opioid poisonings (26). The University of New Mexico Center for Native American Health’s (CNAH) idea for the curriculum arose during this period in response to these disparities. Creating, designing, and updating the curriculum was an ongoing process that drew on ancestral teachings. These symbols communicate cultural knowledge, preservation, spiritual links, and prayers for how the information and visual elements could help Indigenous individuals and communities heal. The CNAH team developed the “Indigenous Cycles of Healing and Recovery Model” based on the Three Sisters model that emphasizes collective growth, mutual benefits, and community health and wellness (27–29).

**Figure 2:**
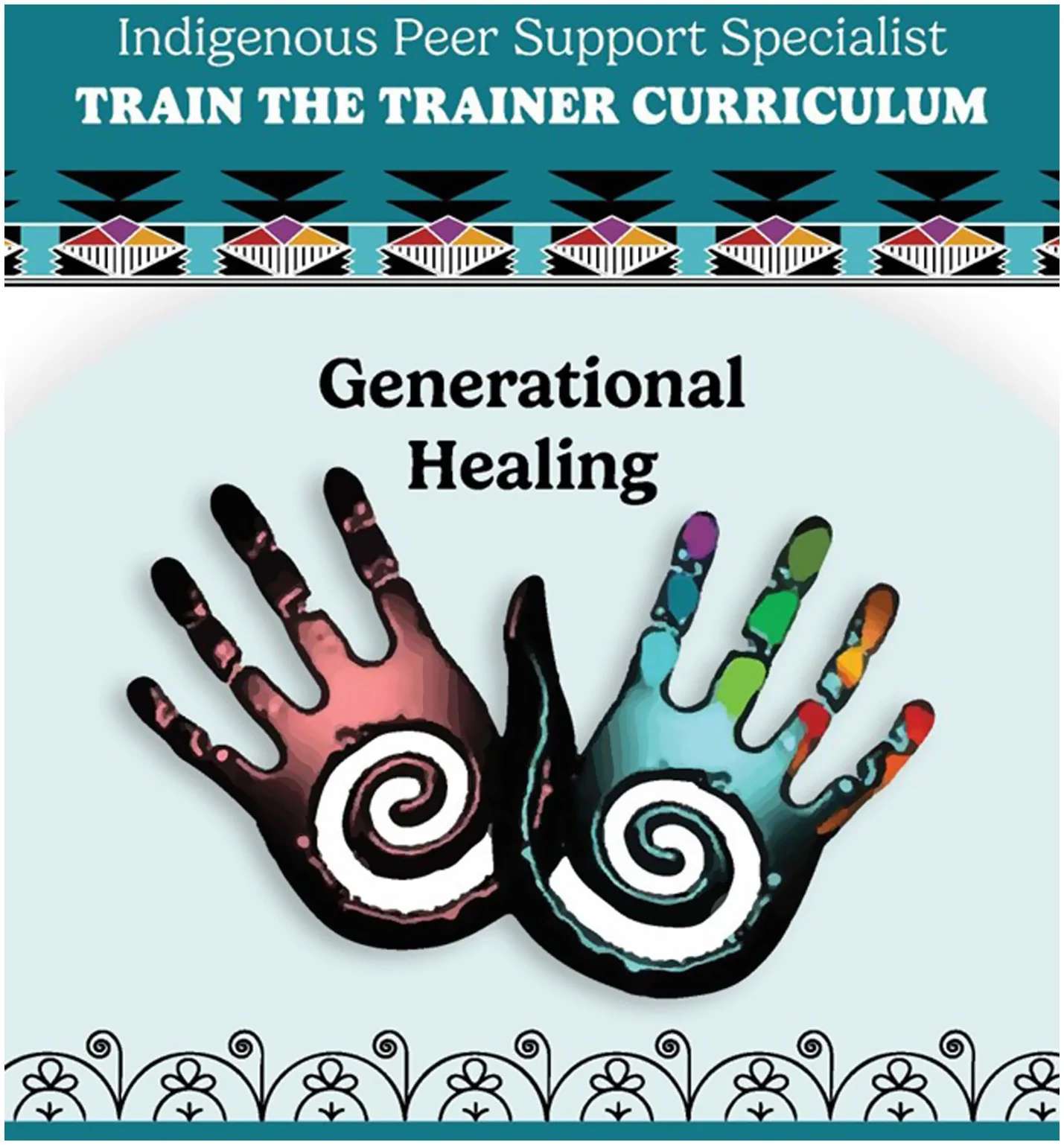
Cover of the Indigenous peer support specialist train the trainer manual.

Each symbol holds special meanings related to healing: the palm represents the physical body, the fingers stand for mental, emotional, and spiritual aspects, and the entire hand signifies human life and energy. The spiral, often seen as the rising sun, can also symbolize evolution, progress, journey, and change. Taken in its entirety, this symbol highlights the importance of connection and support within a community, reflecting the reciprocal relationship between a Peer Support Specialist and those on their recovery path. It also represents dedication to providing comfort and help during tough times. In addition to its role in symbolizing care, the Healing Hand emphasizes healing through traditional medicine and natural remedies, aiming to restore balance and harmony (Figure 2; Table 1).

### 2.5 Curriculum components

The curriculum emphasizes relational learning, reflective practice, and cultural humility, positioning peer support specialists as cultural knowledge holders and facilitators of community-based healing. To strengthen this worldview, the team created an 11-session curriculum that integrates Indigenous values, beliefs, and practices. Recognizing the need to build capacity within Indigenous communities rather than rely on “outside experts,” the team adopted a “train-the-trainer” model. This approach aims to develop local capacity, facilitate knowledge transfer, and ensure sustainability amid high workforce turnover. See Table 2 for curriculum content areas.

**Table 2:** Curriculum content areas.

Title	Content
Part 1: Foundations of peer support	
Session 1 - Beginning	Expectations, agreements
Session 2 - Peer Support functions	Key elements of PRS, types of PRS, efficacy
Session 3 - Principles of recovery	Principles and benefits of PRS
Session 4 - Multiple paths to recovery	Recovery-based support, Indigenous support
Session 5 - Open dialog	Intro, benefits, Indigenous approaches
Session 6 - Western concepts of change	Intro, stages of change, transtheoretical model
Part 2: Foundations of indigenous recovery	
Session 7 - Reclaiming indigenous health/wellness	Intro, theories, Indigenous cycles of healing and recovery model
Session 8 - Historical trauma	Intro, definitions, psychology, impacts
Session 9 - Limitations of medical recovery	Intro, definitions, psychotherapy, existential Indigenous recovery, barriers, alternatives
Session 10 - Cultural humility	Intro, storytelling, patience, confidentiality
Session 11- Relational self-care	Intro, importance of self-care^
Part 3: Vignettes	

Session indigenous cycles of healing and recovery model, conclusion, worksheets. ^Session added based on feedback from participants that additional self-care activities should be included in the curriculum.

At the end of each session, worksheets, interactive activities, prompts, and hands-on learning experiences give participants deeper insights into the content. Sessions accommodate multiple learning styles and preferences, reinforce decolonial teaching methods, and recognize that Indigenous people learn best with culturally centered materials, artistic elements, symbolism, and teachings (30). For example, participants create their own Indigenous Cycles of Healing and Recovery Model using cut-out circles and a metal tab that connects each circle. By locating their current state of recovery within a multi-tiered cultural belief system, participants interact with the model in a tangible way.

### 2.6 Piloting the curriculum

The curriculum was piloted from 2024 to 2026 in partnership with the UNM CNAH team, a peer recovery support worker, and an external community-based recovery organization (AKA). The UNM CNAH team developed a curriculum outline, wrote initial drafts, and shared them with the peer recovery support worker and AKA. The UNM CNAH team’s lived experience of recovery includes healing from trauma. The peer recovery support worker’s lived experiences include 30 + years of sobriety, active engagement in 12-step programs, and work as a peer recovery support worker at a major hospital. The AKA team included authors with the lived experience of healing from substance use disorders. Together, the team met monthly, wrote sections of the curriculum, and reviewed content for culture and application. The team finalized a 143-page manual. The following is a brief timeline of recent events that influenced this curriculum and its development (3, 31).

2020 - The COVID-19 pandemic worsens opioid-related health disparities, and the CNAH team works to identify Indigenous methods of recovery and generational healing through peer recovery support.
2024 - CNAH and AKA critically reviewed content, drawing on existing community-based peer recovery support knowledge and practices from Indigenous communities, and updated grammar, artwork, and visualizations. The cultural significance of hands and symbols is shared and integrated into the culturally centered curriculum. The curriculum was presented to seven elected leaders of a Northeastern Tribal Nation and the supervisor of their peer support specialist program to request Tribal support and advocacy for culturally centered peer recovery support.
2025 - Presented the curriculum at the International Indigenous Ecologies of Love Conference in Santa Ana Pueblo, New Mexico. Delivered a second presentation of the curriculum at the National Indian Health Board Conference in Chandler, Arizona. CNAH facilitates five peer review sessions of the curriculum (each lasting 2 hrs) with five certified Indigenous Peer Recovery Support workers in Albuquerque, New Mexico. CNAH also hosted a seven-hour focus group and talking circle with five Indigenous Peer Recovery Support workers in Albuquerque, New Mexico.
2026 - CNAH reviewed data collected from pilot presentations, analyzed it for meaning-making, planned next steps, and considered implementation in other communities. CNAH also developed the current peer-reviewed article documenting the curriculum’s development and feedback process.

### 2.7 Procedures

Unstructured data was gathered during curriculum development and shared with the team through shared drives, in-person meetings, team meetings, and email. Multiple versions of the curriculum were created, revised, and finalized based on feedback from the CNAH and the consulting team. Activities included in the curriculum were developed and reviewed by an Indigenous Peer Recovery Support community worker. This feedback was crucial for finalizing the curriculum and preparing it for dissemination and presentations. Once finalized, CNAH presented the curriculum at the International Reclaiming Indigenous Ecologies of Love Conference in April 2025, with over 50 attendees, and again at the National Tribal Health Conference in September 2025, with more than 130 participants.

After these sessions, participants were asked to complete an optional online evaluation via Qualtrics. No compensation was provided. To support a thorough, ongoing review, CNAH shared the curriculum with five Indigenous peer reviewers (experts with lived experience) in September 2025 and collected formative feedback through four Zoom meetings. Reviewers were guided by talking-circle prompts aligned with Indigenous knowledge-based principles, ensuring feedback focused on relational validity, community relevance, and practical use.

After these meetings, reviewers were invited to complete an online survey and join a final in-person talking circle to synthesize their feedback. Data from focus groups and talking circles were collected during the meetings. Reviewers gave verbal and written consent to use and share the data and received compensation in line with the cultural value of reciprocity. Data were collected for quality improvement and program development; therefore, they did not require IRB review.

### 2.8 Evaluating and revising the curriculum

**Figure 3:**
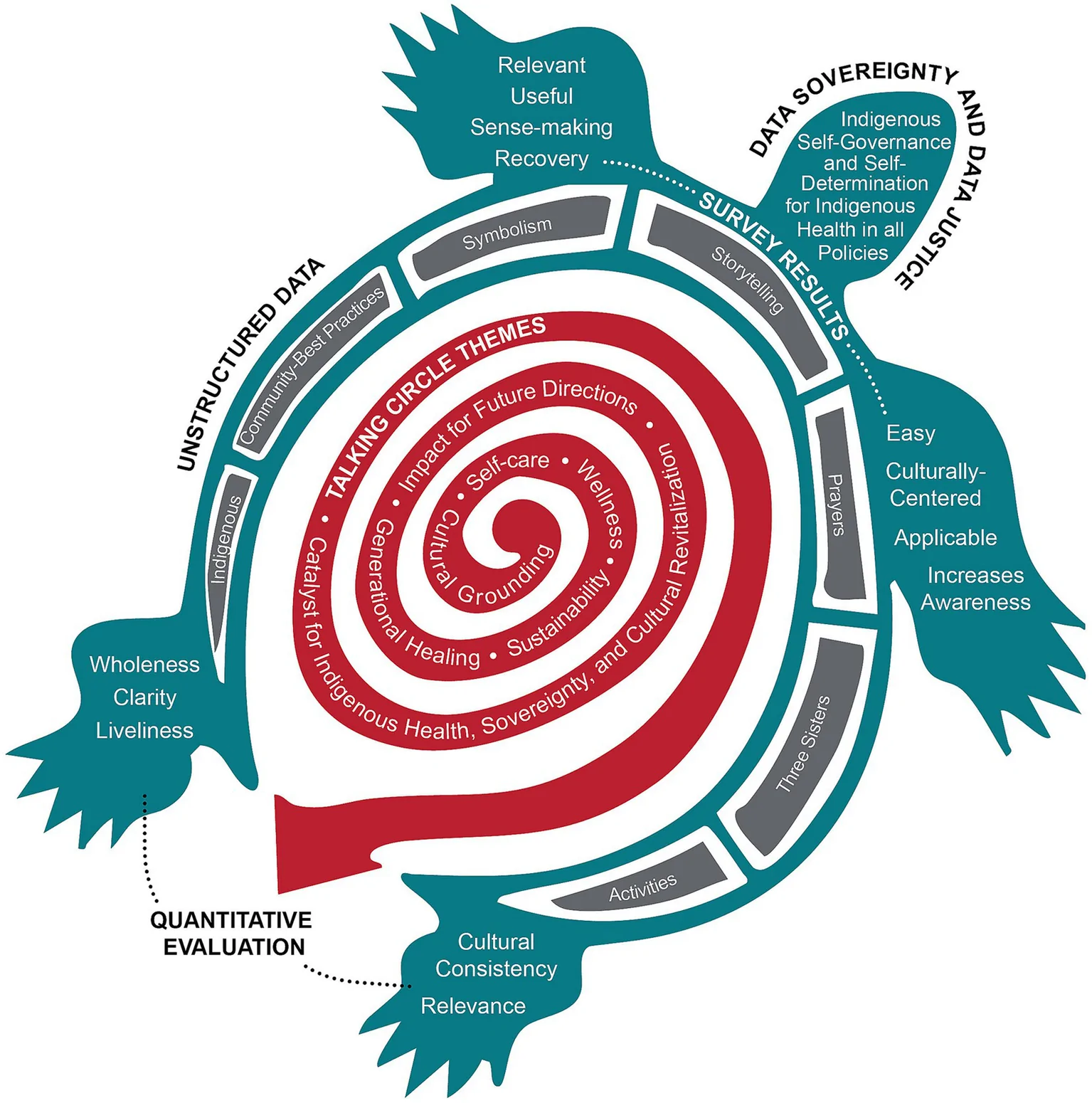
Indigenizing data triangulation on Turtle Island: themes from the Indigenous peer recovery support curriculum formative evaluation.

A culturally centered, Indigenous, mixed-methods, Two-Eyed Seeing approach was used to gather and analyze pilot feedback on the curriculum (32, 33). The “Two-Eyed seeing” approach to healthcare sees with one eye the strengths of Indigenous knowledge and ways of knowing, and with the other, the strengths of Western knowledge, respectfully embracing both (34). We relied on this approach to explore themes from the talking circle participants and qualitative text. We used Western knowledge to analyze and quantify numeric responses from surveys. We also applied the Two-Eyed Seeing approach to develop a process for Indigenizing and triangulating the collected data; we explored how quantitative (Western) and unstructured (Indigenous) data fit together within the contexts of data sovereignty and data justice (Figure 3). Multiple data types were used to explore meaning and identify areas for improvement in training in Indigenous spaces (Figure 3). Table 3 illustrates the Indigenized data triangulation process (see Supplemental File, Data Sheet 3).

**Table 3:** Data triangulation, on Turtle Island.

Data type/source	Data characteristics	Analysis
Unstructured Data	Word documents, literature, and administrative data,	Curriculum revisions and updates based on PRS in AIAN contexts
Survey Results (*N* = 11)	Feedback from conference workshop participants	Mixed-method, frequency counts, thematic analysis
Quantitative Evaluation (*N* = 3)	Feedback from the Peer Reviewer group	Descriptive statistics, means, and standard deviations
Peer-to-Peer Reviewer Focus Group /Talking Circle (*N* = 5)	Semi-structured discussions to evaluate the curriculum	Inductive, Two-Eyed Seeing, thematic analysis

See Supplementary Files 1–4 for a complete list of survey questions, talking circle prompts, and administrative data used.

## 3 Results

### 3.1 Unstructured data and revisions (2024–2025)

Unstructured data was collected and shared throughout the revision process. This included emails with grammar or page edits to the Manual, additional literature reviews to support information in the curriculum, and updates to activities and worksheets based on the CNAH team’s feedback. During the revision, the team also revisited and completed the artwork and graphic designs. Artwork is deeply important to Indigenous communities, as it is closely connected to cultural and knowledge preservation, spiritual connection, artistic expression, and reverence for ancestral wisdom.

### 3.2 Survey results (September 2025)

A total of 11 participants completed an evaluation of the initial piloting of the curriculum at the International Indigenous Ecologies of Love Conference in Santa Ana Pueblo, New Mexico. Most participants (over 82%) felt that this curriculum was useful and relevant to Indigenous communities, that its organization made sense, that its design made it easy to read and focus on what was important, and that the facilitator could easily use it to train others. Over 70% of participants agreed that the length of time (90 min) for this session was reasonable. Most participants (82%) indicated that the curriculum presented would likely bring people into recovery spaces. All participants who completed the evaluation (100%) agreed that the CNAH’s Indigenous Cycles of Healing and Recovery Model was easy to follow and understand, culturally centered, applicable to multiple Indigenous groups, applicable to healing and recovery, and increased awareness of recovery cycles in Indigenous contexts. See Table 4 for the survey results of the curriculum workshop participants.

**Table 4:** Curriculum workshop participants survey results (*N = 11*).

Statement	Response %
The curriculum is relevant to Indigenous communities	82%
This curriculum is useful	73%
The organization/layout of the curriculum makes sense to me	82%
This curriculum will bring people into recovery spaces	82%
The length of time for sessions is reasonable	64%
The design makes it easy to read information and focus on what is important	73%
A facilitator could easily use this curriculum to train others	82%
CNAH’s Indigenous cycles of healing and recovery model is	
Easy to follow and understand	100%
Culturally centered and applicable to multiple Indigenous groups	100%
Applicable to healing and recovery	100%
Increases awareness of the recovery cycles in Indigenous contexts	100%

The response reflects the percentage of respondents who strongly agree or who say “definitely/probably yes”.

Respondents were prompted to provide open-ended responses to several feedback items. When asked about one aspect of the curriculum that they specifically liked, participants reported:

- “The wheel”
- “The models used”
- “The foods as a healing agent”
- “It’s not based on or revolves around a person’s actual age, but where [patients] are actually at in recovery/life.”

One way a participant suggested the curriculum could be improved was by including video testimony from others with personal, lived experience.

Participants were asked what people should know about healing and recovery based on their experiences. They reported:

- “Being patient with others”
- “Meeting others where they are”
- “That healing is possible”
- That the “Indian way is an evidence-based practice with centuries of evidence.”

Participants felt there were several settings where this training could be offered, including Tribal organizations, Tribal recovery facilities, Tribal behavioral health programs, regional and national conferences, reservation-wide, urban Indigenous communities, non-profit organizations, schools, and health care centers.

Others extended appreciation in their Native language: Elahkwa, Nya:weh, Quyana, Lim’lm’tx. Thank you.

### 3.3 Quantitative Indigenous peer reviewer evaluation (October 2025)

Three Indigenous Peer Reviewers were asked to rate each of the 11 in five areas: accuracy, completeness, & wholeness; clarity & readability; cultural consistency; content relevance; and liveliness. Areas were rated on a 1–5 scale from “poor” (1) to “excellent” (5) for 55 total ratings. On average, ratings were “good” (4) or better for 53 of 55 total ratings.

Across the three reviewers and 55 total possible ratings (5 domains, 11 sessions), Session 1 [Beginning] received the highest average rating across items for “content relevance” with a perfect 5.0 “excellent” rating from all respondents. It also received the highest ratings across all domains (see Table 5).

**Table 5:** Indigenous peer reviewer curriculum ratings *(N = 3).*

Session	Accuracy, completeness, and wholeness	Clarity and readability	Cultural consistency	Content relevance	Liveliness
#	M (SD)	M (SD)	M (SD)	M (SD)	M (SD)
1	4.67 (0.58)	4.33 (1.15)	4.67 (0.58)	5.00 (0.00)	4.67 (0.58)
2	4.67 (0.58)	4.67 (0.58)	4.33 (0.58)	4.33 (0.58)	4.33 (0.58)
3	4.33 (0.58)	4.33 (0.58)	4.33 (0.58)	4.33 (0.58)	4.33 (0.58)
4	4.33 (0.58)	4.33 (0.58)	4.33 (0.58)	4.33 (0.58)	4.33 (0.58)
5	4.33 (0.58)	4.33 (0.58)	4.67 (0.58)	4.33 (0.58)	4.33 (0.58)
6	4.33 (0.58)	4.33 (0.58)	4.33 (0.58)	4.33 (0.58)	4.33 (0.58)
7	3.67 (0.58)	3.33 (0.58)	4.67 (0.58)	4.33 (0.58)	4.33 (0.58)
8	4.67 (0.58)	4.33 (0.58)	4.67 (0.58)	4.67 (0.58)	4.33 (0.58)
9	4.33 (0.58)	4.33 (0.58)	4.33 (0.58)	4.33 (0.58)	4.33 (0.58)
10	4.67 (0.58)	4.00 (1.00)	4.67 (0.58)	4.33 (0.58)	4.33 (0.58)
11	4.33 (0.58)	4.33 (0.58)	4.33 (0.58)	4.33 (0.58)	4.33 (0.58)

Ratings on a 1–5 scale from “poor” (1) to “excellent” (5).

Across the 55 total ratings, only two were below 4.0. Session 7 [Reclaiming Indigenous Health & Wellness] received average ratings of 3.3 and 3.7 for “clarity & readability” and “accuracy, completeness, & wholeness,” respectively, reflecting at least one respondent rating it “adequate” (3).

Overall, these ratings indicated strong support for the curriculum’s accuracy and cultural and content relevance. It also highlighted areas for improvement in the clarity and readability of specific sessions. All responding Indigenous Peer Reviewers confirmed that they would use the curriculum with minor revisions.

### 3.4 Peer reviewer group feedback/talking circle august to September 2025

Using an Indigenous-centered qualitative analysis framework, two CNAH team members transcribed the focus group and talking circle recordings. They then conducted an inductive thematic analysis to identify key themes, subthemes, and meaning-making. Transcripts and themes were double-coded, reviewed by the curriculum’s authors, and verified by participants to ensure alignment with lived experience and Indigenous knowledge.

Four overarching domains emerged, reflecting critical aspects of culturally grounded recovery: cultural grounding, self-care, generational healing, and impact and future directions. Definitions for these themes, based on talking circle participant feedback, include the following:

- *Cultural grounding*: Recovery is non-linear, with circular models reflecting Indigenous worldviews. Spirituality is central to healing, and integrating ancestral teachings supports cultural continuity across generations. Recovery is a dynamic process, reflecting the cyclical progression of roles and responsibilities.
- *Self-care, wellness, and sustainability*: The curriculum supports cultural integration in peer practice and frames cultural practices as protective self-care, while seasonal and cyclical self-care models reinforce restoration as culturally embedded and embodied.
- *Generational healing*: Understanding historical trauma as cyclical enables peers to interrupt intergenerational harm and emphasize resilience. Collective storytelling facilitates identity integration and communal healing.
- *Impact and future directions*: Reviewers emphasized the urgency of culturally grounded recovery models and the curriculum’s role in empowering peers, amplifying voices, providing tools to reclaim agency, and embedding culturally grounded lessons into recovery practice.

Table 6 presents illustrative quotes alongside subthemes and meaning-making (see Supplementary file for the full set of themes and quotes). This analysis enabled CNAH to translate reviewer feedback into culturally responsive, practical refinements for peer support specialists in Indigenous communities.

**Table 6:** Qualitative themes from talking circle (*N* = 5).

Main theme	Subtheme	Peer reviewer quotes	Meaning making
Cultural grounding	Spirituality is Central to Healing	“The curriculum’s emphasis on core cultural values and Indigenous spiritual teachings is highly relevant to the recovery process… connecting to the spirit is key to recovery.”	Spiritual connection is positioned as foundational—not supplemental—to recovery, underscoring the inseparability of cultural values and healing processes.
Self-care, wellness, and sustainability	Cultural Integration in Peer Practice	“The curriculum will be helpful for peer support specialists to integrate into their own practice… even for peers who are connecting back to their own culture throughout recovery.”	The curriculum serves as both a professional tool and a personal pathway, enabling peers to sustain their own cultural connection while supporting others.
Generational healing	Historical Trauma as Cyclical	“Thinking about recovery and Historical Trauma lets you see the cycle passed down so you can break it and create capacity for forgiveness. It also showcases the historical resiliency of Native people. Understanding Historical Trauma is very much needed in recovery materials.”	Understanding historical trauma enables peers to interrupt intergenerational harm while reframing recovery through resilience rather than deficit.
Impact and future directions	Urgency for Culturally Grounded Recovery Models	“It would be a disservice not to get this curriculum out into the field immediately. It fills an urgent void in culturally grounded recovery models for Tribal and urban communities.”	Peer reviewers identify a critical gap in recovery infrastructure, positioning the curriculum as a timely and necessary intervention for Indigenous communities.

## 4 Discussion

Results of this pilot study show that CNAH has developed an Indigenous PRS curriculum and has received early participant feedback, highlighting the importance and urgency of providing recovery support services in Indian Country. Participants valued the curriculum’s cultural foundation and its focus on spirituality and healing. This aligns with other research on PRS in AIAN communities, which found that belonging, compassion, and connection are vital to the healing process (35). Self-care, wellness, and sustainability also emerged as key themes from talking circle participants. Here, integrating PRS into daily life is crucial, encouraging peers to maintain their own recovery over time while also gaining from PRS’s broader social support and community benefits. This feedback prompted the development of the final chapter (Chapter 11 of the Manual) on Relational Self-Care. Generational healing and historical trauma are cyclical and require individuals to break these cycles and pursue healing. Talking circle participants emphasized the use of generational healing and forgiveness as central elements in recovery settings. These themes resonate with other research on historical trauma and recovery (36–38). Moreover, PRS in Indigenous communities are inherently beneficial as they provide access to treatment and evidence-based interventions (that are often scarce) in low-resource settings (39).

Although the literature on PRS is limited, future research studies could explore how PRS functions by drawing on concepts like relationality, community connectedness, and cultural grounding (40). Skewes and colleagues’ work on Indigenous Recovery Planning (IRP) interventions shows promise in balancing community capacity, culture, and cultural adaptations to promote community-based healing (41). Continued efforts with the other Indigenous recovery support curriculum, like the Wellbriety curriculum, and the integration of Tribally-specific PRS models are also warranted (20).

### 4.1 Strengths and limitations

This formative evaluation has several strengths and the potential to integrate Indigenous culture into healing spaces by highlighting the lived experiences and voices of our ancestors and future generations. It also demonstrates how traditional stories, language, and symbolism are embedded to enrich the story and tangible experience of participants engaging in recovery and utilizing this healing resource. However, our study has some limitations. First, this is an early-stage evaluation of the curriculum, with various audiences and varying rates of participation. During the April 2025 presentation, the survey link was not working, so none of the participants could complete the survey or provide feedback. During the September 2025 presentation, more than 150 people attended, but only 11 completed the survey. Multiple surveys administered by different groups during the conference likely confused participants and lowered response rates. In the talking circle led by CNAH, only three people completed the quantitative survey. While their responses are valuable, they should not necessarily be generalized to all populations or individuals.

Research to assess the effectiveness of this manual should be conducted by Indigenous researchers working in reciprocity with Tribal Nations and urban Native communities, with healing as the primary goal. There are 576 federally recognized Tribes, each with its own stories, language, symbolism, and healing medicine. The curriculum and teachings were created to be adaptable to Tribal contexts and communities; for example, Tribes can include their traditional language, ceremonies, sources of support, traditional stories, and practices in place of the examples and resources listed in the curriculum. Here, the curriculum is not pan-Indigenous; it is highly adaptable and tailored to Tribal nations, teachings, and practices.

## 5 Conclusion

This formative evaluation suggests that the curriculum is an innovative, culturally rooted intervention that frames Indigenous healing practices as a function of community-based ideologies. By highlighting Indigenous worldviews, relational definitions of healing and recovery, building local capacity, and encouraging sustainable knowledge transfer, this initiative helps promote mental health equity. It may support Indigenous communities in tackling mental health challenges and substance use disorders with dignity, resilience, cultural respect, and sustainability. We carry forward the original teachings and Native languages that have endured throughout time and space. Recovery is conceptualized and arrived at through different methods, but ultimately it means the same thing… To walk in beauty. Love one another. Reflective thought that leads to knowledge and spiritual growth, revealing our purpose on earth. Healing.

## Data Availability

The original contributions presented in the study are included in the article/Supplementary material, further inquiries can be directed to the corresponding author.
