# Supplementary material for: Preliminary findings from a formative evaluation of the Indigenous peer support specialist train-the-trainer manual: a culturally grounded approach to recovery in American Indian and Alaska Native communities
Source: Front Public Health. 2026 Sep 3;14:1833928. doi: 10.3389/fpubh.2026.1833928 (PMC13584764; doi:10.3389/fpubh.2026.1833928)
Supplement: Supplementary file 1 [file Data_Sheet_1.PDF]

HEALTH SCIENCES
CENTER FOR NATIVE AMERICAN HEALTH

Default Question Block

Thank you for completing this evaluation. The Center for Native American Health (CNAH) would like to know more about your experience today. Please respond to the following questions.

How do you feel about what was shared today?

What will you remember in seven days?

How can we improve in the future? Select all that apply.

- ☐ Engage the audience more
- ☐ Use additional visual aids
- ☐ Change the flow of the presentation
- ☐ More time
- ☐ Define recovery and healing terms for shared understanding
- ☐ Less content
- ☐ Different speakers
- ☐ Other, please describe

Please provide any additional feedback about your experience in the space below. Hopefully what you shared will help us improve our presentation in the future. For more information about the manual, visit our landing page and contacts at: https://bit.ly/42zimgC Thank you.

Powered by Qualtrics
