# Supplementary material for: Preliminary findings from a formative evaluation of the Indigenous peer support specialist train-the-trainer manual: a culturally grounded approach to recovery in American Indian and Alaska Native communities
Source: Front Public Health. 2026 Sep 3;14:1833928. doi: 10.3389/fpubh.2026.1833928 (PMC13584764; doi:10.3389/fpubh.2026.1833928)
Supplement: Supplementary file 2 [file Data_Sheet_2.docx]

| **Date** | **Talking Circle Prompt** | **Curriculum Reviewed** | **Primary Review Construct** | **Participants (N)** |
| --- | --- | --- | --- | --- |
| August 27, 2025 | What resonates with you in the curriculum? | Foundations of Peer Support | Relevance with Lived Experience | 6 |
|  | What feels misaligned? | Foundations of Peer Support | Congruence with Community Knowledge |  |
| September 2, 2025 | What session(s) in the manual stood out to you the most? | Principles of Recovery; Open Dialogue; Western Concepts of Change | Meaningfulness within Community Context | 5 |
|  | What resonates with you in the curriculum? | Principles of Recovery; Open Dialogue; Western Concepts of Change | Continuity with Cultural Teachings and Worldview |  |
| September 15, 2025 | What are your thoughts or comments on the material presented in these sessions? | Reclaiming Indigenous Health and Wellness; Historical Trauma | Responsiveness to Community-Identified Priorities | 5 |
|  | In your opinion, how well does the manual address the needs of peer support specialists working in Native American communities? | Reclaiming Indigenous Health and Wellness; Historical Trauma | Usefulness in Supporting Community Roles and Responsibilities |  |
| September 22, 2025 | How did you feel as you reviewed the curriculum’s approach to Indigenous healing and recovery? | Limitations of the Medical Recovery Model; Cultural Humility | Relational Validity | 6 |
|  | What do you expect the impact to be within the communities you work with? | Limitations of the Medical Recovery Model; Cultural Humility | Contribution to Collective Wellbeing |  |
| October 18, 2025 | How different is this from what you currently use in your recovery practice? | Cross-Curricular (Overarching) | Enhancement of Community Strengths | 4 |
| October 18, 2025 | What differences or innovations in this manual stand out to you? | Cross-Curricular (Overarching) | Alignment with Community Knowledge | 4 |
|  | What would you need to feel comfortable using this manual to train others? | Cross-Curricular (Overarching) | Ability to Carry Knowledge |  |
|  | Who should use this manual and how should they use it? | Cross-Curricular (Overarching) | Alignment with Community Roles and Responsibilities |  |
