# Supplementary material for: Preliminary findings from a formative evaluation of the Indigenous peer support specialist train-the-trainer manual: a culturally grounded approach to recovery in American Indian and Alaska Native communities
Source: Front Public Health. 2026 Sep 3;14:1833928. doi: 10.3389/fpubh.2026.1833928 (PMC13584764; doi:10.3389/fpubh.2026.1833928)
Supplement: Supplementary file 3 [file Data_Sheet_3.docx]

Supplement

Figure 3. Indigenizing Data Triangulation: On the Back of a Turtle, Themes from the Indigenous Peer Recovery Support Curriculum Formative Evaluation

Conceptual Triangulation of Data

1. The beginning of the spiral on the turtle's back reinforces the teaching that in Indigenous talking circles there is a beginning point.
2. The front flippers represent the survey results. Once the results are known (+/-) tribal communities use this information to navigate their next moves.
3. The hind flippers represent the quantitative evaluation. Generally, tribal communities are not familiar with quantitative research but embrace qualitative research methods; yet quantitative results become important in moving valuable opportunities forward.
4. The 7 blocks on the back represent the 7 generations that have contributed to the data that is available in various places, hence they represent the unstructured data.
5. Indigenous data sovereignty and data justice are another aspect of this curriculum’s outcome. Such opportunities allow tribes to control how they will provide holistic and wellbeing for their community members from their unique cultural standings.
