# Supplementary material for: Preliminary findings from a formative evaluation of the Indigenous peer support specialist train-the-trainer manual: a culturally grounded approach to recovery in American Indian and Alaska Native communities
Source: Front Public Health. 2026 Sep 3;14:1833928. doi: 10.3389/fpubh.2026.1833928 (PMC13584764; doi:10.3389/fpubh.2026.1833928)
Supplement: Supplementary file 4 [file Data_Sheet_4.docx]

**UNM CNAH Peer Support Specialist Training at the NIHB National Tribal Health Conference**

Indigenous Peer Support Specialist Train-the-Trainer Manual:

Embracing Culturally Responsive Recovery

September 9, 2025

The UNM Center for Native American Health hosted a session at the National Indian Health Board2025 Conference that provided a comprehensive overview of the Indigenous Peer Support Specialist Train-the-Trainer Manual (IPSSTTM), highlighting its role in addressing the mental and behavioral health crises intensified by COVID-19 within Indigenous communities. Participants engaged with the cyclical Indigenous Cycles of Healing and Recovery Model and explored its contrasts to traditional Western recovery models. Emphasis was placed on building local capacity through peer support training, integrating Indigenous cultural practices, and ensuring the sustainability of mental health initiatives.

A total of 11 participants completed an evaluation of this event. Most participants (over 82%) felt that this curriculum was useful and relevant to Indigenous communities, that the manual's organization made sense, that the design made it easy to read and focus on what was important, and that the facilitator could easily use the curriculum to train others. Over 70% of participants agreed that the duration of this session was reasonable. Most participants (82%) indicated that the curriculum presented would bring people into recovery spaces.

All participants who completed the evaluation agreed that the CNAH's Indigenous Cycles of Healing and Recovery Model was easy to follow and understand, culturally centered, applicable to multiple Indigenous groups, and relevant to healing and recovery. They also reported that it increased awareness of recovery cycles in Indigenous contexts.

Participants were asked to give open-ended responses to several items. When asked about one aspect of the curriculum they particularly liked, participants mentioned: the wheel, the models used, the foods as a healing agent, and that “It’s not based on or revolves around a person’s actual age, but where [patients] are actually at in recovery/life.” One way a participant thought the curriculum could be improved was by adding video testimonies.

Respondents were asked what people should know about healing and recovery based on their experiences. They reported being patient with others, meeting people where they are, that healing is possible, and that the “Indian way is a[n] evidence-based practice with centuries of evidence.”

Participants were also asked where the “Indigenous Peer Support Train the Trainer” could be offered. Responses included interest in having this training within their organization, at tribal facilities, on reservations, within tribal schools, at other conference presentations, and within behavioral health and other clinical settings.

**Appendix A- Frequency of Responses**

The curriculum is relevant to Indigenous communities

- Strongly agree = 9 (82%)
- Somewhat disagree = 1 (9%)
- Strongly disagree = 1 (9%)

This curriculum is useful

- Strongly agree = 8 (73%)
- Somewhat agree = 1 (9%)
- Somewhat disagree = 1 (9%)
- Strongly disagree = 1 (9%)

The organization/layout of the manual makes sense to me

- Strongly agree = 9 (82%)
- Somewhat disagree = 2 (18%)

This curriculum will bring people into recovery spaces

- Strongly agree = 9 (82%)
- Somewhat disagree = 1 (9%)
- Strongly disagree = 1 (9%)

The length of time for sessions is reasonable

- Strongly agree = 7 (64%)
- Somewhat agree = 1 (9%)
- Neither agree nor disagree = 1 (9%)
- Somewhat disagree = 2 (18%)

The design makes it easy to read information and focus on what is important

- Strongly agree = 8 (73%)
- Somewhat agree = 1 (9%)
- Strongly disagree = 2 (18%)

A facilitator could easily use this curriculum to train others

- Strongly agree = 9 (82%)
- Somewhat disagree = 1 (9%)
- Strongly disagree = 1 (9%)

**CNAH's Indigenous Cycles of Healing and Recovery Model is…**

Easy to follow and understand

- Definitely yes = 9 (82%)
- Probably yes = 2 (18%)

Culturally centered and applicable to multiple Indigenous groups

- Definitely yes = 9 (82%)
- Probably yes = 2 (18%)

Applicable to healing and recovery

- Definitely yes = 10 (91%)
- Probably yes = 1 (9%)

Increases awareness of the recovery cycles in Indigenous contexts

- Definitely yes = 10 (91%)
- Probably yes = 1 (9%)

**What spaces and places do you envision the Indigenous Peer Support Train the Trainer being offered?**

- In our organization
- At tribal facilities
- Behavioral Health
- All conferences regarding human services, law, etc.
- Reservations. Urban indigenous communities, non-profits
- In schools as an evidence-based practice
- Santo Domingo pueblo or Kewa Pueblo Health Corporation
- I want to offer this training in California to tribal health programs
- Health care center

**What is one aspect of the curriculum that you like?**

- The infancy to elder categories
- Models
- Everything
- People place themselves and not diagnosis or labels
- It’s not based on or revolves around a person’s actual age, but where they are actually at in recovery/life
- The foods as a healing agent
- Indigenous
- The wheel

**What is one thing that you would improve in the curriculum?**

- Add video testimony

**What do people need to know about healing and recovery based on your experiences?**

- Be patient
- Meet them where they are at.
- You don’t stay at one area, and you go back and forth
- It is possible
- That the Indian way is a practical, evidence-based practice with centuries of evidence
- Asking for help

**Please provide additional feedback in the space below. Thank you for your time. For more information, contact Dr. Tassy Parker, TaParker@salud.unm.edu**

- It was interesting
- Quyana- Thank you
- Lim'lm'tx. Thank you.
